# Implementation of a Children's Safe Asthma Discharge Care Pathway Reduces the Risk of Future Asthma Attacks in Children–A Retrospective Quality Improvement Report

**DOI:** 10.3389/fped.2022.865476

**Published:** 2022-03-29

**Authors:** Lesley Kennedy, Gillian Gallagher, Barbara Maxwell, Brigitte Bartholme, Andrew Fitzsimons, Catherine Russell, Orla Mallon, Jenny L. Hughes, Susan Beattie, Veena Vasi, Dara Bartholomew O'Donoghue, Michael David Shields

**Affiliations:** ^1^Royal Belfast Hospital for Sick Children, Belfast Health and Social Care Trust, Belfast, United Kingdom; ^2^School of Medicine, Dentistry and Biomedical Science, Queen's University Belfast, Belfast, United Kingdom; ^3^Paediatric Department, Antrim Area Hospital, Antrim, United Kingdom

**Keywords:** asthma attack, discharge care pathway, Teach-to-goal, inhaler technique, understanding action plan

## Abstract

**Background:**

Many children attend Emergency Departments (ED) and Out of Hours (OoH) frequently for acute asthma. Follow up care is often suboptimal leaving these children at risk of a future attacks. We report on the development, implementation and evaluation of a safe asthma discharge care pathway (SADCP).

**Methods:**

This is a retrospective report on the development, implementation and evaluation of outcomes of a SADCP. The pathway was based on the Teach-to-goal educational methodology that supported the mastery correct inhaler technique and ability to action the personalized asthma action plan (PAAP). Children with frequent asthma attacks were entered as they were discharged from the Emergency Department or ward. The first training session occurred within 1–3 weeks of the index asthma attack with 2 further sessions in the following 8 weeks. Children exiting the pathway were discharged either back to primary care or to a hospital clinic.

**Results:**

81 children entered the pathway (median age 5 years) with 72 discharged from the ED and 9 from the medical wards of the Royal Belfast Hospital for Sick Children. At pathway entry 13% had correct inhaler technique, 10% had a Personalized Asthma Action Plan (PAAP), and 5% had >80% (45% >50%) repeat refill evidence of adherence to inhaled corticosteroid over the previous 12 months. On pathway exit all children demonstrated correct inhaler technique and were able to action their PAAP. One year later 51% and 95% had refill evidence of >80% and >50% adherence. Comparisons of the 12 months before and 12 months after exit from the pathway the median number of emergency ED or OoH asthma attendances and courses of oral corticosteroids reduced to zero with >75% having no attacks requiring this level of attention. Similar findings resulted when the SADCP was implemented in a district general hospital pediatric unit.

**Conclusion:**

Implementing an asthma care pathway, using Teach-to-Goal skill training methods and frequent early reviews after an index asthma attack can reduce the future risk of asthma attacks in the next 6 to 12 months.

## Introduction

An asthma attack in the previous year is a predictor of a future asthma attack (1, 2). A significant percentage of children are re-admitted within a short time period after having an asthma attack which required hospitalization. In-addition, recurrent asthma attacks is a common reason for multiple crisis attendances at the Emergency Department (ED) (3, 4). The British Thoracic Society (BTS) asthma guidelines and National Review of Asthma Deaths (NRAD) report recommend that adults and children with asthma are seen in primary care within 1–2 days of an acute attack and by 4 weeks with a specifically trained asthma specialist in Secondary Care (5, 6). The NRAD reported that 10% of asthma deaths occurred within 28 days of hospital discharge and 21% had attended the ED in the previous year with more than half of these having attended more than once. Therefore, the NRAD report made the strong recommendation that follow-up arrangements should be made after every attendance at an ED or out-of-hours (OOH) service. Secondary care follow-up should be arranged after every hospital admission for asthma and for patients who have attended the ED two or more times with an asthma attack in the previous 12 months. Thus systems should be in place to facilitate the appropriate movement of patients from the acute setting into an asthma care program (6–8). Red flags for the prediction of potential asthma deaths in the NRAD report included; (a) incorrect inhaler technique, (b) non-adherence to preventer therapy, (c) absence of a Personalized Asthma Action Plan (PAAP), and (d) on-going exposure to asthma triggers. The basics of good asthma care that address these red flag are not in place for more than half of asthma patients in N Ireland and in even fewer in mainland Great Britain (9).

The regular use of inhaled corticosteroids taken with correct inhaler technique forms the pharmacological backbone of asthma therapy and ensuring the basics of asthma care are in place should bring both better day-to-day asthma control and reduce the risk of a future attack for most children (10). PAAPs typically state which treatments are required when the child is: (1) stable (e.g., regular adherence to preventer medication, Green zone), (2) action which should be taken at the start of an exacerbation or when asthma control has deteriorated (e.g., a head cold with increased coughing and the start of wheezing, Amber zone), (3) how to manage an acute attack of asthma (red zone). Having an up-to-date PAAP as part of supported self-management of asthma has previously been shown to be beneficial (11). It is important to ensure understanding of, and the ability to action, the written PAAP (green, orange, red zones) as well as understanding when and why to take each medication.

The Royal Belfast Hospital for Sick Children (RBHSC) provides tertiary pediatrics for N. Ireland (population approximately 1.9 million) and acute secondary care for the children of Belfast with typically 20–30 acute asthma admissions each winter month. On average 180 children with acute asthma attacks attend the ED in each of the winter months and more than 50 in each of the summer months. In 2009 the Public Health Agency of the Department of Health Social Services N Ireland (PHA, DHSSNI) conducted an audit of all the N Ireland E.D.s (*n* = 14) and 7 of the 8 acute Out of Hours (OoH) facilities. In this audit, covering a 2 week period, 14% of all attendances were for asthma attacks and about 20% of these cases were for repeat visits. Despite being advised to, only 8% of the adults and children attended their GP for follow up assessment within 2 weeks after the asthma attack (unpublished data) thus missing the opportunity to ensure good asthma preventative measures are in place. In addition, the RBHSC took part in an annual British Thoracic Society audit of acute asthma care. Like most other units we performed satisfactorily with the in-hospital acute management but we underperformed in the discharge process which was targeted at providing the child with a management programme (12). Children with frequent asthma attacks that require admission, attendance at the ED or OoH for crisis management are a group that requires more attention. An asthma attack is an important point of entry into an asthma care program so that the basics of asthma care can be implemented (7, 8).

We here report (retrospectively) on a Safe Asthma Discharge Care Pathway (SADCP) that we developed, implemented and then evaluated at the RBHSC and then applied the SADCP to a local children's District General Hospital unit.

## Methods

The PHA DHSSNI, as part of the “Transforming Your Care” initiative, agreed to facilitate the development of the children's Safe Asthma Discharge Care Pathway (SADCP) for N Ireland.

The SADCP aimed to use the acute asthma attack presentation at ED (with or without hospital admission), the index attack, as an opportunity to ensure the basics of asthma care were put in place for each child. It was planned to pilot and refine the pathway at both the RBHSC and at a local District General Hospital (Antrim Area Hospital, JLH, SB) and if beneficial then to have it rolled out to all pediatric units in N Ireland.

This is a retrospective narrative description of the development, implementation and service evaluation of our Safe Asthma Discharge Care Pathway. We include audits and checks on the outcomes and report the overall outcomes using a before/after design. The project was approved by the Standards Quality & Audit Department of the Belfast Health & Social Services Trust and was not deemed research (13).

### Background Initiatives and Knowledge That Informed the Pathway Development

In 2012 we invited 52 consecutive children who were either being discharged after a hospital admission (*N* = 40) or who were frequent ED attenders (≥4 ED attendances for asthma attacks in the previous year, *N* = 12) to a new specialist asthma nurse (SB, GG) clinic. Twelve required more appropriate inhalers prescribed, all children were trained to use inhalers correctly at that clinic appointment (using the iterative Teach Back or Teach to Goal (TB/TTG) method, Box 1) (14–16). All children were given an updated written PAAP along with asthma education with emphasis on the need for adherence to the inhaled corticosteroid. We learned from this; (a) that the post discharge clinic should be held between 1 and 2 weeks following the index asthma attack (as all children attended) and (b) the nurses needed further follow up educational sessions in order to be sure the basics of asthma were being correctly implemented. Evidence suggests that one off educational sessions are inadequate and support the need for several educational sessions (17–19). In addition, we previously reported that children who had been trained to use inhalers correctly made critical errors while using their inhalers at home. Using remote video directly observed therapy (vDOT) it took up to 3 weeks with daily feedback before all children studied were able to demonstrate correct inhaler technique on a regular basis (20). Recent studies which used the TB/TTG teaching method showed that, while this technique is much better than simply demonstrating correct inhaler technique, the beneficial effects wane with patients relapsing back into incorrect inhaler techniques when assessed 4 weeks later (15, 16).

Box 1 Teach Back/Teach-to-Goal (TB/TTG) teaching technique. After iterative training on both correct inhaler technique and actions required to implement the PAAP zones (green, amber, and red), each child and their parent were required to retrieve from memory, teach back and demonstrate both correct inhaler technique and understanding of PAAP on 3 separately spaced occasions within each consultation.

**Table:** 

	**Inhaler technique**	**Ability to action PAAP (green, amber, red zones)**
Initial Iterative training	On correct inhaler technique	Actions required to implement the PAAP zones
Teach back	Child/parent to explain back to trainer each step and reason why it is needed.	Explain back action required for each PAAP zone
Demonstrate back	Demonstrate that child can do each step correctly	
Repeat the above steps correctly on 3 separate occasions within each individual teaching session		

Following this initial pilot of a new nurse led asthma discharge clinic we believed that in order to ensure both mastery of correct inhaler technique and to ensure understanding and ability to correctly action the PAAP (for the green, amber and red asthma control zones, supported self-management) that we needed the following;

1) to make a follow up educational appointment soon after an index asthma attack.
2) to use the TB/TTG teaching method not only for training children to correct inhaler mastery but also to ensure their ability to action their PAAP (Box 1).
3) to provide 3 educational sessions 2–3 weeks apart in the 8–9 weeks post discharge after the index asthma attack. Several such teaching sessions (*N* = 3, each applying memory retrieval and spaced learning TB/TTG) aimed to address the waning over time of the learned skills (14–19, 21).

## Phase 1–Development of the Safe Asthma Discharge Care Pathway

A series of multidisciplinary team (MDT) meetings were arranged with representative children and their parents, primary and secondary care asthma nurses, general practitioners and pediatricians from both ED and respiratory medicine along with facilitators from the PHA, DHSSNI.

At the MDT meetings the following decisions were made;

### Who to Target the Care Pathway

We agreed the following criteria for referral to/entry into the Safe Asthma Discharge Care Pathway (Supplementary Material 1).

- >1 years age (to avoid overlap cases with recurrent or persistent bronchiolitis symptoms).
- Children <15 years (the upper age that the RBHSC ED department accepted cases).
- Children with a doctor asthma diagnosis (those currently on preventer asthma medication).
- include those with frequent (<3) episodic viral wheeze episodes and currently treated with ICS and classic atopic asthma.
- >1 previous visit to A&E or hospital admission with wheeze in the previous 6 months.

First time wheezing episodes were therefore not to be included in this initial pilot period.

### When Should the Children Be First Seen at the Asthma Nurse Clinic?

The MDT decided not to follow the BTS recommendation that children are seen within 1–2 days of discharge–as this was currently unachievable and likely the clinic visit would concentrate on whether the attack had completely resolved. In addition, the recommended 4 week specialist review was considered too long a delay to wait as it was considered that the parental worry surrounding the acute attack may have faded increasing the possibility of reduced attendance. Given the excellent attendance at our initial pilot clinic (described above) review between 1 and 2 weeks following the index attack was considered as the optimal time.

### Ensuring Immediate Safe Discharge From the ED or Wards and Referral to the Nurse Led Asthma Clinic

Given the intense workload and changing staff in the ED department an immediate safe discharge from ED department pathway proforma was developed. The aim being to prevent an early re-attendance during the next few weeks before the child could be seen at the asthma clinic. The child/parent would be informed that an appointment with a specialist asthma nurse would be organized between 1 and 2 weeks' time.

The same was true for discharge after a ward admission. Although the same asthma nurses (running the new pathway asthma clinic) may have had an opportunity to start the education process prior to ward discharge. Children are rapidly discharged once they no longer require oxygen and their breathless has subsided but often well before all wheezing has cleared up. Immediate discharge checklist proforma were developed (Supplementary Materials 2A,B) for the ED and hospital wards respectively. The same proforma was used as a referral document to the pathway Asthma Nurse and a printed copy given to the parents.

Each day the asthma nurse would collect the ED and ward referrals from the previous day and arrange the clinic review via the appointments office who sent out a letter inviting the child and parent to attend at the next available nurse led clinic in 1 and 2 weeks following their discharge.

### What Was to Be Included in the Nurse Led Asthma Intervention of the SADCP and Why

We agreed that for this intervention to be effective the educational clinics needed to include the following features;

- Standard explanation of the nature of asthma, triggers and the roll of each type of medication (background education) with emphasis placed on the need for regular preventer inhaled corticosteroids (ICS) therapy irrespective of current symptoms.
- Training to mastery of inhaler technique using the Teach Back (or Teach to Goal) methodology (TB/TTG).
- Training to full understanding of the PAAP also using the TB/TTG approach–so that child and/or parent should be able to explain back to the asthma nurse the actions required for each of the green, amber and red zones in the PAAP.
- Each child should be offered 3 educational sessions over a 6–8 week period following the index asthma attack.
- Once mastery of the required skills had been achieved the asthma nurse would make the decision based on current asthma control to either request a] follow up by Primary Care or b] follow up at one of hospital based asthma clinics.

To allow time for comprehensive instruction, 45 min was allocated to new referral patients and 30 min for each follow-up review.

**Figure 1:**
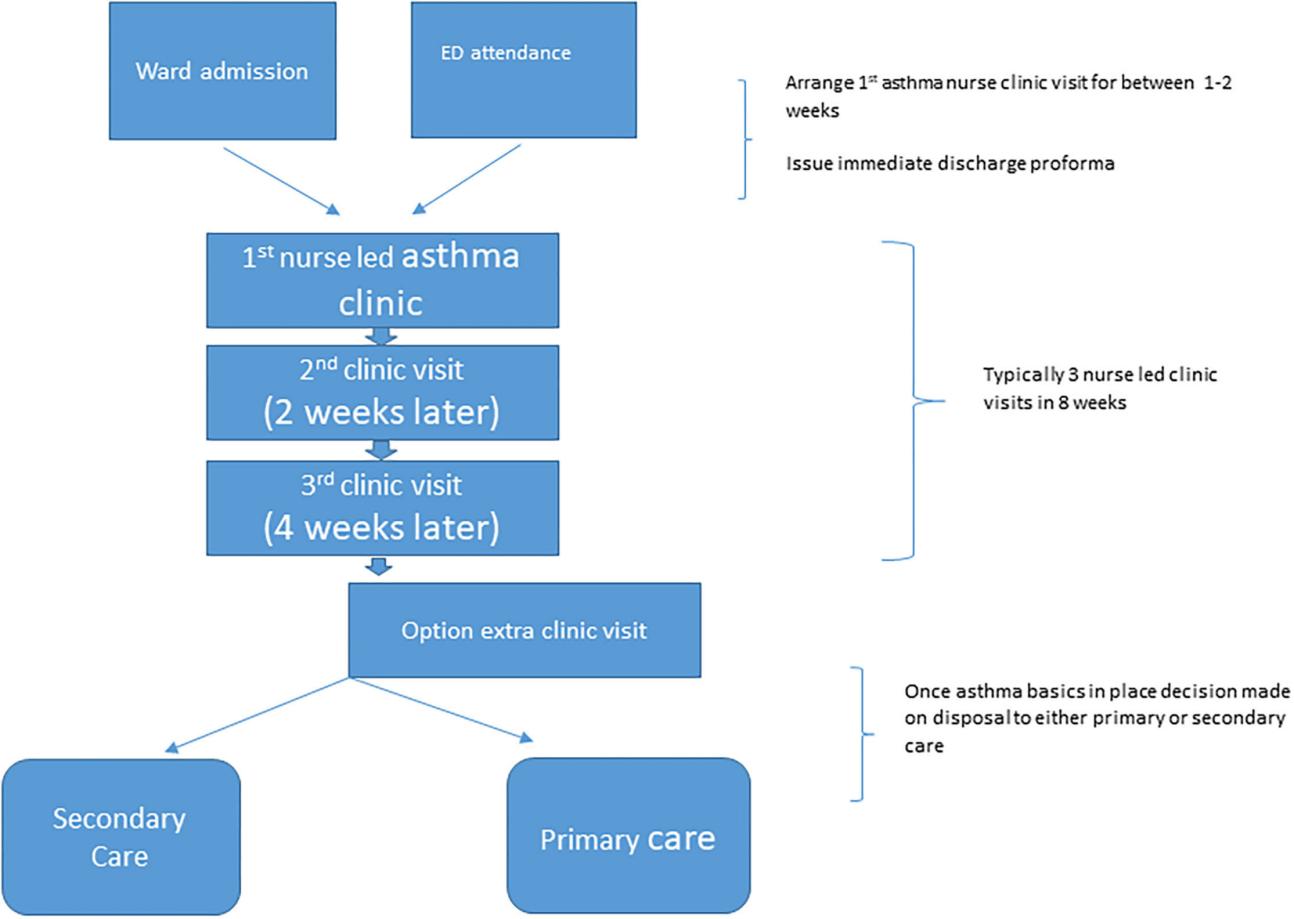
The final SADCP summary algorithm.

Figure 1 summarizes the final agreed Safe Asthma Discharge Care Pathway.

## Phase 2. Implementation of the Safe Asthma Discharge Care Pathway

### Ensuring Correct Patients Were Referred and Attended the Pathway Asthma Clinic

Asthma nurses introduced an ongoing system to educate medical and nursing staff rotating through ED and the medical wards regarding the Care Pathway. Initially there was a problem with both under and inappropriate referral. In order to improve the referral rate of appropriate cases every morning the consultant (BB, AF) in ED checked the entries for all the previous 24 h attendances and identified any potential missed cases, this acted as a safety net to ensure all suitable children had been referred. Inappropriate referral (mostly children with problem cough who were queried as having asthma or first time wheezers) was addressed by further education of the ED staff.

To help ensure families of children attended the asthma clinics they were told that they would be contacted and invited to a full and detailed asthma review on a specified date and time in the next 1 to 2 weeks. The importance of attendance was stressed explaining that the aim was to reduce the chances of their child having another acute attack.

### Barriers and Issues Occurring During Implementation

(1) Prior to implementation of the pathway the most commonly used systemic oral corticosteroid used was prednisolone, however, during the implementation there was a transition to using single dose dexamethasone as the preferred oral steroid. The immediate discharge care pathway (Supplementary Materials 2A,B) therefore was changed to include Dexamethasone and local General Practitioners were informed that a second dose dexamethasone could be given to those whose symptoms had not adequately resolved after 2 days.

(2) Incompatibility of electronic records. Originally, it was planned that the immediate discharge checklist proforma for ED (Supplementary Materials 2A,B) would be placed on each child's N Ireland Electronic Record (NIECR) so that those in primary and secondary care would have access. However, it was not possible to link the ED department record with the NIECR and therefore paper copies were required to be printed. The child's pathway documentation and updated PAAP were therefore first uploaded onto NIECR at the time of the first pathway asthma clinic visit by the nurse.

(3) Teach Back or Teach to Goal (TB/TTG) methodology was used to ensure children had mastered correct inhaler technique and this required the use of trainer inhalers or placebo devices. The RBHSC organization does not allow the re-use of trainer inhalers or holding chamber devices (single patient use) and an adequate and ongoing supply of trainer/placebo inhalers needed to be obtained from Pharmaceutical companies. Hospital stock small volume holding chambers and, if used in training, were given to the child for future use.

## Phase 3-Evaluation of the Safe Asthma Discharge Care Pathway as a Service Improvement

The PHA,DHSSNI requested that the final pathway (Figure 1) should be piloted and results audited prior to any decision regarding potentially rolling out the pathway across all N Ireland's pediatric units and consideration of modifying the pathway to suit adult asthmatics.

The clinically collected data was recorded in a fully anonymised spreadsheet for further analysis. We carried out the service evaluation to determine the impact of this SADCP once it had been established at both the RBHSC and Antrim Area Hospital pediatric units.

### Data Collected

The numbers of child/parents engaging with the SADCP were recorded as well as the number dropping out. The number of asthma educational sessions required before the nurses considered that the “asthma basics” were correctly in place was recorded.

At the start of the clinic session the nurse assessed the child's current inhaler technique and understanding of their PAAP. Assessment of the inhaler technique was made using the manufacturer's checklist for each inhaler type.

The nurse classified each child's inhaler technique using a global assessment (20):

- “Correct” (all important steps carried out and nurse formed the opinion that the child would have received most of the expected dose).
- “Partial” (in which case the child made errors but the nurse felt that the child could have received at least some but not most of the dose).
- “Poor” technique (where critical errors were being made and the nurse formed the impression that likely little or none of the dose would be delivered to the lungs).

The nurse recorded whether the child already had a PAAP and if so assessed their understanding of this.

The N Ireland Electronic Care Record (NIECR) was used to record the acute asthma health care utilization of the first 81 children who were referred to and entered into the pathway and who had at least 12 months of follow up care (either in primary care or secondary) after exiting from the Safe Asthma Discharge Care Pathway. The number of acute courses of oral corticosteroids used, attendances at OoH and ED and any hospital admissions for acute asthma were recorded. In addition, an estimate of adherence to the inhaled preventer (ICS) was calculated from refills prescribed over the 12 months before and the 12 months following the child exiting the pathway. Before and after comparisons were made for the 12 months before with the 12 months after the index acute asthma attack for all children. We repeated the before/after analysis excluding those aged under 4 years who might be expected to have reduced wheezing over time.

On a separate sample of children currently on the pathway, an independent observer (OM) checked whether the child/parents had mastered the correct inhaler technique and had the ability to action the PAAP (green, amber, red zones) when leaving a pathway clinic visit.

A similar evaluation of a consecutive convenience sample of those entered into the care pathway at Antrim Area Hospital pediatric unit (the selected District General Hospital) was conducted by obtaining similar data on care pathway children for the 6 months before and after exiting the pathway.

#### Results of the Evaluations

The first 81 consecutive patients for whom 1 year of time had elapsed since exiting the SADCP at the RBHSC were studied. The median age was 5 years (IQR: 3 to 6 years, maximum 13 years). The majority were referred from ED (*N* = 72, 89%) and 9 were referred following a hospital admission. Those children/parents failing to attend the first appointment were telephoned to offer a new appointment and all children had attended by 3 weeks. Every child attended at least once with 10 having one single visit, 19 children had two clinic sessions and 37 had 3 sessions. For thirteen of the 19 children having only two clinic visits the third visit was deemed unnecessary as the nurses considered the children/parents had already mastered the asthma basics. However, 6 of the 19 did not attend further despite repeat phone calls. Unexpectedly, for sixteen children/parents the nurses considered they needed greater than the expected 3 training sessions and these children continued to be seen at the nurse led clinic until it was considered the skills could not be improved with further training.

Thirty-five children were classified as having a frequent episodic viral wheezing (43%) pattern while 46 fitted the multi-triggered wheeze pattern typically with day-to-day milder wheezing interspersed with acute attacks. Thirty-two had clinical atopy (mostly concomitant Allergic Rhinitis and /or atopic eczema). At baseline all children had previously been prescribed anti-asthma treatment with 34 on the BTS asthma guideline Step 2 and 47 on BTS Step 3.

Surprisingly in this cohort, few had obvious known trigger factors that could be easily avoided. Only 6 reported coming from homes where one or other parent smoked cigarettes, 6 had homes with damp and mold and 5 had pets which from the clinical history avoidance should have been considered. Advice was given about trigger avoidance whenever appropriate.

### Improvements Made to Child's Care During the Safe Asthma Discharge Care Pathway

**Figure 2:**
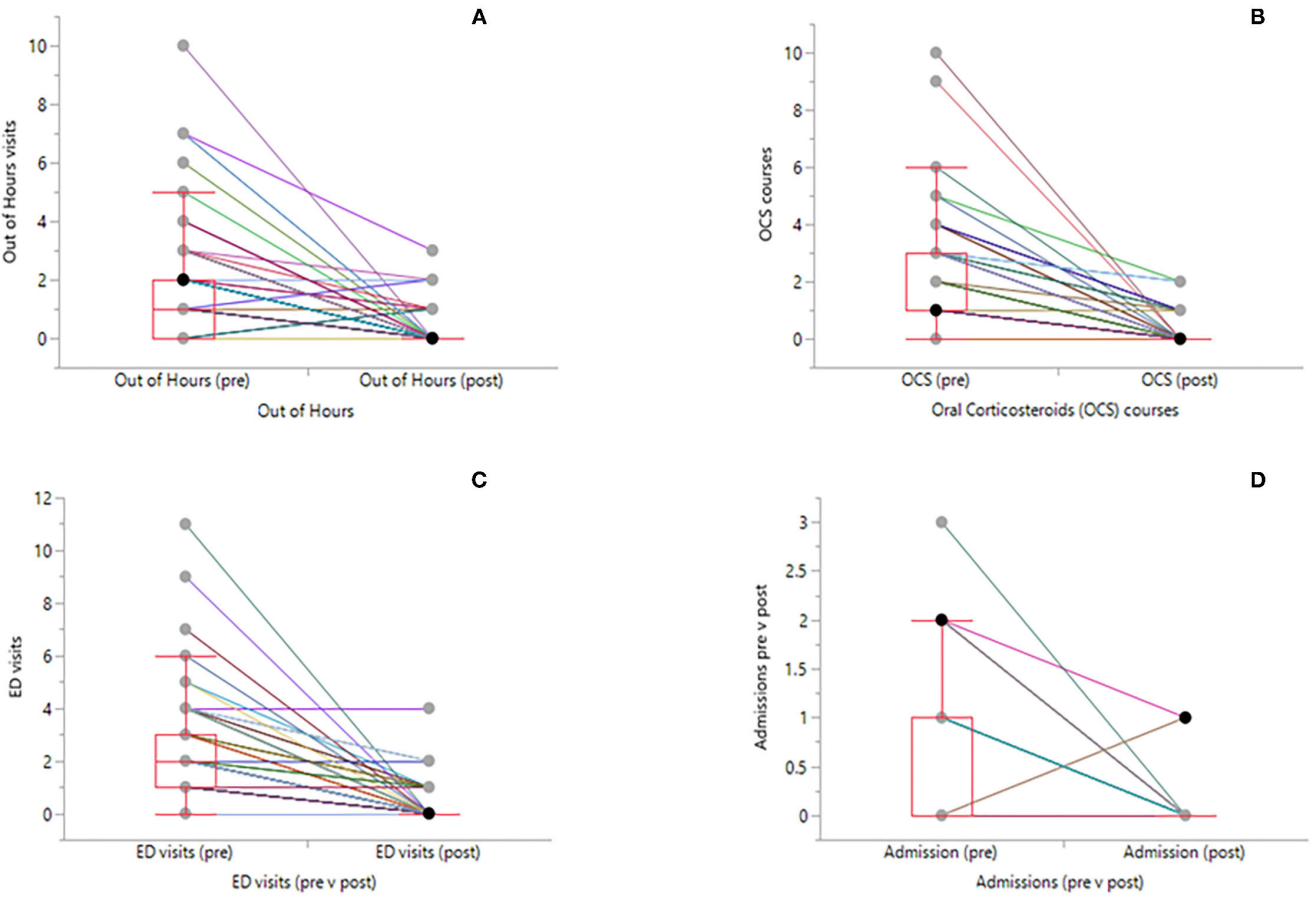
Before and after Box plots with individual patients linked by solid lines. On the y-axis are number of OoH visits **(A)**, number of courses OCS **(B)**, number of ED visits **(C)** and number of admissions **(D)**. The center horizontal line in the box is the median, the upper horizontal box lines are the quartiles and dots above the upper whisker represent individual outliers.

At the start of the Nurse led intervention only 13 of the 81 (16%) children had correct inhaler technique, 38 had partially correct technique and the remainder (*N* = 30) had poor technique and many of these required a change to a more age appropriate inhaler device. At baseline eight of the 81 (10%) children had evidence that they had been given a PAAP (Table 1). In the 12 months prior to the index asthma attack only 4 (5%) out of the 81 had evidence from prescription refills of >80% adherence (average calculated inhaler use based on refills collected over the previous 6 or 12 months) and for 33 (41%) the adherence was between 50 and 80%. At the end of the SADCP educational sessions all 81 were deemed to have mastered correct inhaler technique and had a good understanding of how to action their PAAP. In the 12 months post the intervention 42 (52%) of the 81 had evidence for GP prescriptions refills suggesting >80% adherence and 36 (44%) had refill adherence of between 50 and 80% (Table 1). The effectiveness of the pathway was demonstrated by statistically significant reductions in courses of OCS, attendances at out of hours and Emergency department visits and admissions for acute asthma (Table 2, Figures 2A–D). These improvements were observed for children both older and younger than 4 years.

**Table 1:** Changes made during the nurse led training sessions at the RBHSC.

	**Baseline, on entry to care pathway**	**At end of pathway nurse led asthma intervention**
**Inhaler technique (** ***N*** **)**		
Poor	30	0
Partial	38	0
Correct	13	81
**Possession of up to date PAAP (** ***N*** **)**		
No	73	0
Yes	8	81

**Table 2:** For the RBHSC.

	**12 months Pre-intervention**	**12 months Post-intervention**	
**Oral corticosteroids courses**
Median (IQR, range)	1 (IQR: 1–3, range: 1–10)	0 (IQR: 0–0, range: 0–4)	*P* < 0.0001
**Out of Hours attendance**
Median (IQR, range)	1 (IQR: 0–2, range: 0–10)	0 (IQR: 0–0, range: 0–2)	*P* < 0.0001
**ED attendances**
Median (IQR, range)	2 (IQR: 1–3, range: 1–11)	0 (IQR: 0–0, range: 0–4)	*P* < 0.0001
**Admissions**
Median (IQR, range)	0 (IQR: 0–1, range: 0–3)	0 (IQR: 0–0, range: 0–1)	*P* < 0.0001
**Adherence to ICS (** ***N*** **)**
<50%	44	4	
50–80%	33	36	
>80%	4	41	

*Number of courses Oral Corticosteroids, Number of Out of Hours attendances, number of ED attendances and number of admissions for acute asthma attacks in the previous 12 months compared with the 12 months following the asthma nurse led intervention. The Wilcoxon paired test was used to assess whether this before/after improvement was statistically significant (P < 0.05). Adherence to inhaled corticosteroids (ICS) was calculated from primary care refills*.

#### Check of the Effectiveness of the Training Given to Child/Parents Attending at the Pathway Clinic

An independent assessor (OM) evaluated a separate convenience sample of children immediately before and again after leaving one of the pathway clinics. This aimed to determine if the children had mastered correct inhaler technique and whether the children/parents were able to explain what action they needed to take when the child's asthma control put them in the PAAP green, amber and red zones. The assessor sampled 26 child/parent units after their initial first clinic visit within the pathway and assessed a further 42 children who were having their 2^nd^ or 3^rd^ review. The assessor recorded inhaler technique steps and then formed a global assessment according to the 3 categories (correct, partial, poor technique) for both preventer and reliever inhalers. The results are presented in Table 3. We checked for understanding of, and the ability to action, the given written PAAP after the same children/parents as above. We found that only 11 of the 26 (42%) of new patients attending their first visit on the care pathway already had a written PAAP. All the review patients had been given a PAAP but only 14 brought the PAAP to the review clinic making it difficult to confirm whether they had a written PAAP. After leaving the clinic all child/parent patients were able to describe what the medications did and when to use them. In addition, all patients were able to correctly describe what medications to use when the child's asthma control was in both the green and red zones of the PAAP. A number of patients were unclear regarding action to take when the child was entering the amber zone e.g., onset of a head cold and mild increase in cough and / or wheeze. Overall, this check audit of the nurse led clinics confirmed that using the TB/TTG technique for both training in mastery of correct inhaler technique and understanding of and ability to action the PAAP was working.

**Table 3:** For the RBHSC.

	**Total number (before training)**	**Total number (after training)**	**New (before training)**	**New (after training)**	**Review (before training)**	**Review (after training)**
**Preventer**	
Correct	29	61	9	23	20	38
Partial	29	6	9	3	20	3
Incorrect	10	1	8	0	2	1
**Reliever**	
Correct	24	59	6	24	18	35
Partial	30	8	10	2	20	6
Incorrect	14	1	10	0	4	1

*Global inhaler technique before and after TB/TTG inhaler education for both new and review children*.

#### Implementing the Safe Asthma Discharge Care Pathway in a District General Hospital Pediatric Unit (Antrim Area Hospital Children's Unit)

The SADCP was evaluated at the children's unit of Antrim Area Hospital (JLH, SB). Antrim Area Hospital is a District General Hospital (DGH) with a pediatric and neonatal unit which covers the local area of County Antrim in N Ireland but which refers cases to the tertiary regional centre at the Royal Belfast Hospital for Sick Children.

We selected a convenience sample of 27 children who had been through the care pathway at least 6 months before so that we had data on the 6 months before and 6 months after exit from the care pathway. This included 14 children with multi-triggered atopic asthma and 13 children with episodic viral wheeze all of whom had previously been prescribed inhaled corticosteroids. Twenty-four children entered the pathway after an asthma ward admission and 3 were referred from the ED department. After completing the Care pathway 15 children were referred back to primary care and 12 referred to the local hospital asthma clinic. Four (15%) children entering the care pathway already had a written PAAP and 23 did not. All were given an updated PAAP after the first clinic visit. Five of the 27 patients only attended one clinic visit, 14 attended two clinics, 6 attended 3 clinics and 2 required 4 clinic visits. The outcomes of the before and after requirements for OCS, OoH attendances, ED attendances, admissions and calculated adherence to asthma preventer medication are summarized in Table 4.

**Table 4:** For AAH.

	**6 months Pre-intervention**	**6 months Post-intervention**	
**Oral Corticosteroids Median (IQR, range)**	1 (IQR: 1–2, range: 0–8)	0 (IQR: 0–1, range: 0–2)	*P* < 0.001
**Out of Hours attendance**			
Median (IQR, range)	0 (IQR: 0–1, range: 0–4)	0 (IQR: 0–0, range: 0–1)	*P* = 0.03
**ED attendances**
Median (IQR, range)	2 (IQR: 1–2, range: 1–4)	0 (IQR: 0–0, range: 0–1)	*P* < 0.001
**Admissions**
Median (IQR, range)	1 (IQR: 0–1, range: 0–3)	0 (IQR: 0–0, range: 0–1)	*P* < 0.001

*Describes the number of courses Oral Corticosteroids, Number of Out of Hours, number of ED attendances and number of admissions for acute asthma attacks in the previous 6 months compared with the 6 months following the asthma nurse led intervention. The Wilcoxon paired test was used to assess whether this before/after improvement was statistically significant (P < 0.05)*.

## Discussion

We have developed and implemented a Safe Asthma Discharge Care Pathway (SADCP) that utilizes the index acute asthma attack as a window of opportunity to carry out and deliver an asthma management programme to children. This SADCP meets the recommendations suggested in the NRAD report (6). At least for the children exiting the SADCP the evaluation suggest this should reduce the risk for a future asthma attack, however, a full randomized controlled trial would be needed to confirm this. In addition, the benefits applied equally to those managed at the tertiary referral children's hospital and at a District General Hospital unit.

There are some important aspects of the asthma nurse led interventions within the SADCP that are worthy of emphasis. Firstly, we ensured that parents were aware that, shortly after discharge (from the ED or ward after the child's asthma attack), they would be invited to an arranged clinic appointment with the asthma nurse–the reason given was that we wanted to reduce the risk of a future attack. Informing and pre-arranging the follow up appointment may have led to better child attendance.

Secondly, we ensured that adequate time was set aside for the nurse led clinical sessions. We have observed that many children with asthma children receive frequent acute crisis care but are only offered short (e.g., 10 min) appointments in either primary or secondary care once or twice a year for their asthma reviews. We believe that it is impossible to do a comprehensive asthma assessment and provide training to mastery of inhaler skills in such short consultations.

Thirdly, we believe that use of the TB/TTG teaching methodology was important. Simply showing (brief instruction) a child/parent how to use an inhaler does not mean that correct inhaler technique will be carried out at home (21, 22). A recent systematic review of inhaler technique studies report no improvement for each of the last 4 decades despite improvements in inhaler device design (about 30% of asthmatics have correct technique) (23). Studies of using the TB/TTG technique show that this is a very do-able method of teaching within an asthma clinic setting, however, the beneficial effects wane by 28 days. In cognitive psychology it is recognized that re-testing and retrieval, as demonstrated by teach back are important to consolidate learning (24–27). The three clinic visits in 8 weeks help to reinforce memory retention through what has been called “spaced repetition.” We provided 3 educational visits within an 8 week period in our SADCP asthma programme. Several studies have shown that 3 separate teaching sessions are needed typically 2–3 weeks apart to provide sustained good inhaler technique (17, 18). The effectiveness of spaced repetition in creating long-term memories and attenuating memory decay has been experimentally demonstrated in long-term memory formation (24–27). Finally, we extended the use of TB/TTG methodology to ensure that children/parents have a good understanding of how to utilize their PAAP. This is important because children experience asthma attacks even when the asthma basics of care are being correctly applied. Children and or parents were able to describe what action to take when the child's current asthma was in the green or red zones of the PAAP but some were less clear with what action to take at the start of an asthma exacerbation (amber zone). The reasons for this may include the numerous different individual scenarios that may not have been discussed and that the nurses themselves may not have had a clear view of specific action needed given this area has a conflicting evidence base (e.g., conflicting evidence regarding doubling the inhaled corticosteroids).

Our SADCP asthma management programme, like others, has multiple components and we unable to determine whether all aspects of the SADCP would be essential to have made similar improvements. However, from our experience and knowledge of the literature we applied the following; (1) utilized the acute attack as the window of opportunity, (2) ensured timely first entry into the SADCP i.e., between 1 and 2 weeks, (3) ensured each patient had adequate time with the asthma nurse, (4) used TB/TTG teaching methodology and (5) reviewed the child to provide re-inforcement of learning on 3 occasions in an 8 week time frame. We believe the SADCP as a package resulted in the improved outcomes for these children.

Although we have not performed a formal cost-benefit analysis the major extra costs required included in setting up this Safe Asthma Discharge Car Pathway were (1) having trained asthma nursing staff, and (2) allocating the asthma nurses with the clinic time (45 min per patient), equipment (e.g., single patient demonstration inhalers) and clinic space. The administration of the system was easily integrated into the work performed by the current administrative staff. Given that we found a large increase in preventer asthma medication (ICS and /or combination ICS+ beta2 agonist) adherence the cost of asthma medication would be an additional extra cost to the Health Service (prescriptions for children in N Ireland are free). The cost savings with in the hospital would be more difficult to calculate. The small reduction in ED attendances (e.g., 195 instead of 200 daily attendances) is only likely to result in reduced patient waiting times and a minimal drug cost saving (salbutamol plus oral steroid). Asthma attacks are one of the most feared consequences of having asthma and clearly the benefits to children and their families in reduced asthma attacks and potentially reduced risk of asthma death is self-evident and hard to cost.

## Limitations

This is a before and after service improvement evaluation report. While the improvements in children's asthma outcomes seen are most likely due to the implementation of the asthma education programme a causal link cannot be confirmed. This would have required a Randomized Controlled Trial. It is well known that asthma varies over time and especially between seasons. However, as we recruited the children over a 1 year period, it is likely we would have equally caught children going from their bad to good season and vice versa. Younger children are more likely to have a transient episodic viral wheeze that resolves over time meaning that some improvements were due to natural resolution. The children in our study who had a symptom pattern in keeping with episodic viral wheeze had all previously been started on ICS suggesting that they were suffering frequent episodes. We found similar statistically significant improvements when we excluded those aged under 4 years from the analysis.

We did not collect data on the children's day to day asthma control over the period studied but only indices of acute asthma attacks and therefore we cannot comment on whether each child's day-to-day symptom control was better. In addition, we were unable to find out whether, at 12 months post intervention, the parents had acted on the trigger avoidance advice suggested. We were surprised that fewer than expected children came from households with a parental smoker, how few houses were reported as having damp and mold and how few children had household pets to which they were allergic. Trigger avoidance is stressed in recent asthma guidelines but health care professionals can be uncertain as to how effective exclusions of an allergen can be and can feel that the efforts involved can be a futile exercise. In addition, there is very little information available on child / parent beliefs about trigger avoidance and motivation to make changes. This is an area that needs further research.

Finally, the recent Coronavirus (COVIT-19) pandemic has accelerated the use of Tele-Medicine as lockdowns and mandates meant that families and staff were less keen on face to face clinical meetings unless they were absolutely essential. While we believe face-to-face training is optimal it has been reported that inhaler instruction using the Teach-to-goal/Teach back technique is eminently possible via remote video consultation (28). Our SADCP therefore would lend itself to hybrid approaches with perhaps the first clinical assessment being face-to-face followed by some video consultations. Further research is needed into determining whether our care pathway could be further improved and streamlined using the combination of video consultations and video Directly Observed Therapy.

## Conclusions

We found that implementing a nurse led safe asthma discharge care pathway (SADCP) following a child's acute asthma attack was feasible in both a major children's hospital and in a District General Hospital children's unit and for the children was followed by a reduced number of asthma attacks in the following year. Our SADCP could be modified and applied to adults after an asthma attack and also should be considered in primary care.

## Data Availability Statement

The original contributions presented in the study are included in the article/Supplementary Materials, further inquiries can be directed to the corresponding author.

## Ethics Statement

Ethical review and approval was not required for the study on human participants in accordance with the local legislation and institutional requirements. Written informed consent from the participants' legal guardian/next of kin was not required to participate in this study in accordance with the national legislation and the institutional requirements.

## Author Contributions

MS and JLH conceptualized and initiated the process for the development of the care pathway. LK, SB, BM, MS, JLH, BB, VV, and AF were the health care professionals (asthma, respiratory and emergency medicine) involved with the pathway development. LK, SB, GG, and CR delivered the care pathway. LK and SB collated the recorded data. OM and DO'D, performed the independent audit of the children's mastery of inhaler technique. MS analyzed the data. MS, JLH, DO'D, VV, and LK edited and wrote the initial draft manuscript. All authors have approved the paper.

## Funding

The Public Health Agency (PHA) of the Department of Health, Social Services N. Ireland (DHSSNI) facilitated the development and initial implementation of Safe Asthma Discharge Care Pathway.

## Conflict of Interest

The authors declare that the research was conducted in the absence of any commercial or financial relationships that could be construed as a potential conflict of interest.

## Publisher's Note

All claims expressed in this article are solely those of the authors and do not necessarily represent those of their affiliated organizations, or those of the publisher, the editors and the reviewers. Any product that may be evaluated in this article, or claim that may be made by its manufacturer, is not guaranteed or endorsed by the publisher.

## Abbreviations

ED: Emergency Department
OoH: Out of Hours
SADCP: Safe Asthma Discharge Care Pathway
DGH: District General Hospital.
