# Supplementary material for: Implementation of a Children's Safe Asthma Discharge Care Pathway Reduces the Risk of Future Asthma Attacks in Children–A Retrospective Quality Improvement Report
Source: Front Pediatr. 2022 Mar 29;10:865476. doi: 10.3389/fped.2022.865476 (PMC9001987; doi:10.3389/fped.2022.865476)
Supplement: Supplementary file 1 [file Data_Sheet_1.docx]

**Supplement 1**

**ROYAL BELFAST HOSPITAL FOR SICK CHILDREN**

**Nurse-led Asthma Clinic Referral Protocol for ED**

**PLEASE ENSURE EVERY PATIENT REFERRED IS GIVEN A SAFE FOR HOME CHECKLIST**

**AIMS:**

1. To reduce hospital admissions
2. To reduce the frequency of ED attendances
3. To reduce acute asthma attacks
4. Reduce number of OCS in a year

**OBJECTIVES:**

1. To improve inhaler technique
2. To improve compliance
3. To ensure adequate education on self-management of asthma to the child and parent
4. To ensure follow-up by the hospital or GP

**CRITERIA FOR APPOINTMENT:**

**ACUTE ASTHMA**

Children over 1 year old and under 15 years old presenting with an acute attack to ED, on a second occasion including this one within the previous 6-months.

**EPISODIC VIRAL INDUCED WHEEZE (VIW)**

Children > than 1 year old with VIW already using regular ICS or who have had > 3wheezing episodes in the previous 6 month period.

**OR**

Children with a confirmed diagnosis of asthma who nursing and medical staff feel would benefit from further education on asthma management.

**EXAMPLES OF THOSE NOT FOR REFERRAL:**

1. Children presenting with acute bronchiolitis
2. Children with good background control and inhaler technique but have attended with an attack due to an URTI.
3. First episode or infrequent Viral Induced Wheeze not on any treatment.

**HOW TO MAKE A REFERRAL:**

- Complete ED referral form
- Provide a copy of ED flimsy and place in asthma folder provided
- Inform parents that they will receive an appointment through the post
- Asthma nurse will arrange the appointment

**CONTACT DETAILS FOR ASTHMA NURSES:**

**Supplement 2a Immediate discharge checklist proforma for ED.**

**Supplement 2b. Immediate discharge checklist proforma for ward discharges.**
